# Interference with Protease-activated Receptor 1 Alleviates Neuronal Cell Death Induced by Lipopolysaccharide-Stimulated Microglial Cells through the PI3K/Akt Pathway

**DOI:** 10.1038/srep38247

**Published:** 2016-12-02

**Authors:** Yuxin Li, Wuyang Yang, Alfredo Quinones-Hinojosa, Baocheng Wang, Shujun Xu, Weijie Zhu, Feng Yu, Shaoji Yuan, Peigang Lu

**Affiliations:** 1 Department of Neurosurgery, Jinan General Hospital of PLA, Jinan, Shandong, 250031, P.R. China; 2 Department of Neurosurgery, Johns Hopkins University School of Medicine, Baltimore, MD, 21205, USA; 3 Department of Neurosurgery, Qilu Hospital, Shandong University, Jinan, Shandong, 250000, P.R. China

## Abstract

Excessive microglial cells activation in response to inflammatory stimuli leads to synaptic loss, dysfunction, and neuronal cell death. Activated microglia are involved in the pathogenesis of neurological conditions and frequently contribute to several complications. Accumulating evidence suggests that signaling through PAR-1 is involved in inflammation, however, its function has yet to be fully elucidated. Here, we have demonstrated that the suppression of PAR-1 leads to down-regulation of inflammatory factors including IL-1β, IL-6, TNF-α, NO, as well as the prevention of activation of NF-κB in BV2 cells. In addition, we found that a PAR-1 antagonist, SCH, prevented LPS-induced excessive microglial activation in a dose-dependent manner. As a result of SCH treatment, neuronal cell death via up-regulation of Akt-mediated pathways was reduced. Our results demonstrate that the beneficial effects of SCH are linked to its ability to block an inflammatory response. Further, we found that SCH inhibited the death of PC12 neurons from the cytotoxicity of activated BV2 cells via activation of the PI3K/Akt pathway. These neuro-protective effects appear to be related to inhibition of PAR-1, and represents a novel neuroprotective strategy that could has potential for use in therapeutic interventions of neuroinflammatory disease.

Microglial cells are the resident innate-immune cells of the central nervous system (CNS), and play critical roles in immune surveillance and host defense by acting as the primary responding cells in the CNS^1^. Emerging evidence demonstrates that excessive microglial-mediated inflammation in the brain leads to synaptic loss, neuronal dysfunction and cell death. In disease, these effects contribute to pathogenesis and progression of various CNS diseases conditions incuding stroke^2^, infection^3^, neurodegeneration, brain trauma, pain^4^,^5^,^6^, tumors, and other insults to the CNS^7^.

When healthy brain tissue becomes lesioned, microglia cells notably shift their morphology from a thin and ramified to large and amoeboid^8^. In a healthy response, these cells normally engage protective cytokines such as IL-10, which suppresses inflammation and promotes neuronal repair^9^,^10^. When microglia are chronically activated, they may cause neuronal injury through the release of proliferative and cytotoxic molecules, such as NO and pro-inflammatory cytokines (IL-6, TNF-α, and IL-1β)^11^. Unfortunately, work on specific molecular and cellular pathways between microglia and neurons is limited. Among this work, one important transcriptional factor is NF-κB, which requires phosphorylation of IκB as a prerequisite for its activation, has been described. NF-κB is involved in regulating expression of proinflammatory mediators, including cytokines, chemokines, and adhesion molecules, thereby playing a crucial role in mediating inflammatory responses^12^,^13^.

Previous studies have also demonstrated that thrombin, a pluripotent serine protease plays a critical role in hemostasis and coagulation. Thrombin is known to have inflammatory functions via G protein-coupled receptors, also known as protease-activated receptors (PARs), inculding protease-activated receptor 1 (PAR-1)^14^. PAR-1 has been implied in a variety of CNS physiological processes depending on the activate serine protease and its target cell type^15^. Signaling through PAR-1 appears to mediate the process of brain damage in adult animal models of subarachnoid hemorrhage (SAH)^16^,^17^. Previous rodent studies have shown that the interference with this pathway in primary astrocyte cultures^18^,^19^ as well as in hippocampal slice cultures^20^ provided a potential target for therapeutic approach in maintaining vascular integrity following brain damage in rats^21^,^22^.

While the effect of PAR-1 activation on cell survival and proliferation has been demonstrated in microglia^15^, little is known regarding its role in modulating microglia-mediated inflammatory responses effects on neural cells. This study is designed to elucidate **1)** the cross-talk between BV2 microglial activation and PC12 neuronal death. **2)** whether SCH imposes an anti-inflammatory effects against lipopolysaccharide (LPS)-stimulated inflammatory responses in BV2 cells to protect PC12 neurons, and **3)** the downstream effectors involved. To isolate PAR-1’s role in this process, a PAR-1 antagonist termed SCH79797 (SCH)^23^ was utilized. Additionally, we discovered that a key signaling enzyme implicated in cell survival^24^, phosphatidylinositol 3-kinase (PI3K), is involved in the protection offered by PAR-1. Finally, the combined effect of LY29004 (Ly), a PI3K/Akt inhibitor^25^, and SCH was also investigated.

## Results

### Effect of LPS Stimulation and SCH Treatment on Morphological Changes of BV2 Microglia

**Figure 1:**
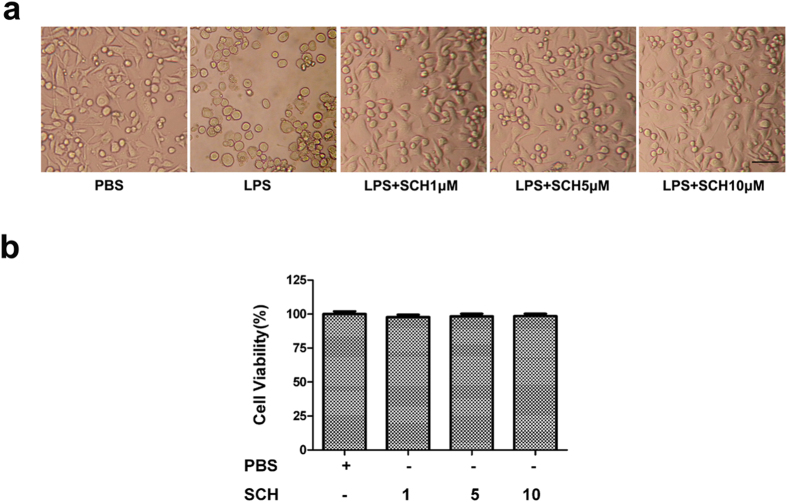
Morphological changes of LPS-stimulated and SCH-treated BV2 microglia (**a**), and its viability after exposure to SCH alone (**b**). Scale bar = 200 μm.

We first investigated the impact of LPS and SCH stimulation on the morphology of BV2 microglia. In the control group, BV2 microglia displayed small soma with long distal arborization. In contrast, LPS-stimulated BV2 microglia exhibited larger and rounder shape with retracted branches. BV2 cells treated with 1 μM SCH showed fewer, shorter branches that appeared to be reasorbed into the soma of the cells. Cells treated with 5 μM SCH showed moderate improvement in cell branch morphology. BV2 microglia treated by 10 μM SCH showed obvious morphological improvement on the retraction of cellular projections, the shape of cell body also recovered from larger and round to a slender appearance (Fig. 1a). To determine the toxicity profile, cytotoxicy measurement was taken using MTT assay. SCH did not show any toxicity in BV2 cells with the concentrations being 1, 5, 10 μM (Fig. 1b).

### The Expression of PAR-1 in BV2 Microglia

**Figure 2:**
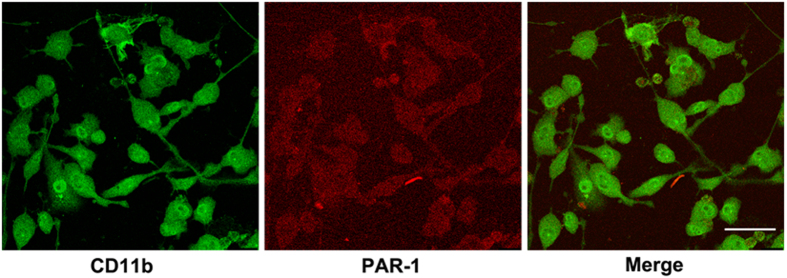
The expression of PAR-1 in BV2 microglia. Immunofluorescence images of PAR-1 and a microglia marker CD11b. Cell bodies and processes of rat microglia showed strong immunoreactivity of CD11b (green) and PAR-1 (red). Superposition of the two images was also shown. Scale bar = 100 μm.

Cultured BV2 microglia were double-labeled with microglial cell markers, CD11b and PAR-1, which showed clear co-localization using fluorescent probes as described above. Cell bodies and processes of BV2 microglia labeled with the antibodies to CD11b (green) and PAR-1 (red) displayed expression throughout the entire cell. Merging channels also showed that the two markers were almost exactly co-localized, and were present throughout the cell (Fig. 2).

### Effect of SCH on the Production of Inflammatory Cytokines in Activated BV2 Microglia

**Figure 3:**
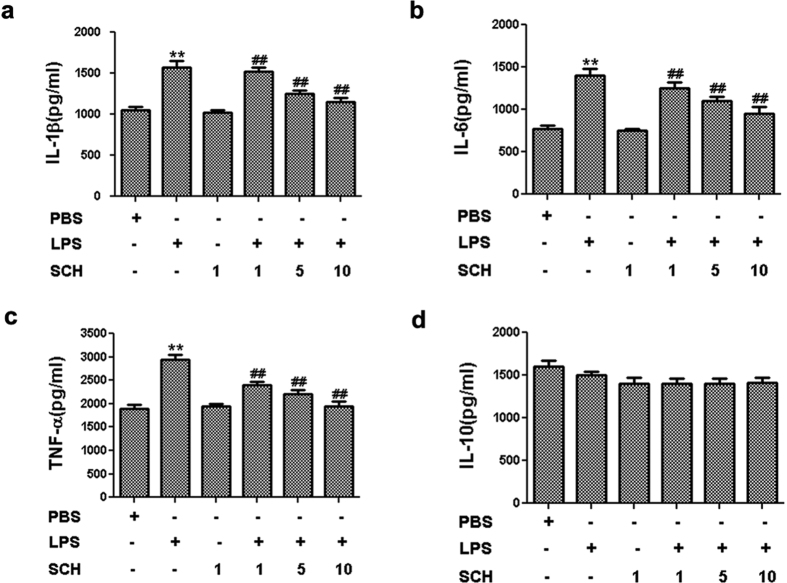
Effects of SCH on IL-1β, IL-6, TNF-α, and IL-10 production in LPS-stimulated and SCH-treated BV2 cells. After treatment for 24 hr, ELISA was used to analyze the release of IL-1β (**a**), IL-6 (**b**), TNF-α (**c**), and IL-10 (**d**) production from the supernatants, respectively. Data were expressed as mean ± SD (n = 3). **p < *0.001* versus control; ^##^*p* < *0.01* versus LPS (1 μg/ml) group.

**Figure 4:**
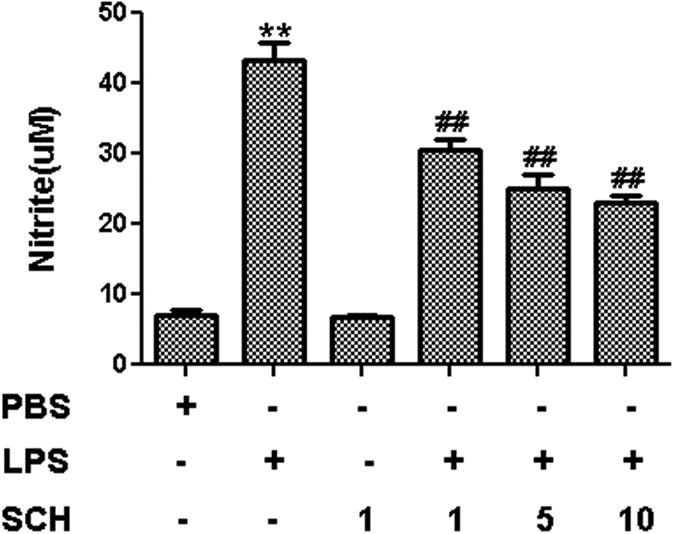
Effects of SCH on NO production in LPS-stimulated and SCH-treated BV2 microglia. The supernatant were isolated to determine the NO production by Griess reagent, respectively for 24 hr. Data were expressed as mean ± SD (n = 3). **p < *0.001* versus control; ^*##*^*p* < *0.01* versus LPS (1 μg/ml) group.

In order to confirm any suppressive effects against neuro-inflammation, the effect of SCH on the production of proinflammatory cytokines IL-1β, IL-6, TNF-α, and IL-10 and inflammatory mediator NO in activated BV2 microglia were investigated. BV2 microglia were treated with LPS (1 μg/mL) for 24 hr, co-incubated with 1, 5, and 10 μM SCH, respectively, and then changed into fresh medium for another 24 hr to get conditioned medium. ELISA and Griess reagent assays were used to determine the cytokine levels and the amounts of nitrite, which represent the levels of NO, from activation of BV2 microglia. The results showed that LPS alone could dramatically promote the production of IL-1β, IL-6, TNF-α, and NO in the supernatant (Figs 3a–c and 4, *p* < *0.01*). Compared with control group, there were no significant changes in the secretion of anti-inflammatory cytokine IL-10 (Fig. 3d, *p* > *0.05*). However, compared with those in the LPS group, co-incubation with 1, 5, and 10 μM SCH for 24 hr markedly decreased the levels of IL-1β, IL-6, TNF-α, and NO in the supernatant from LPS-treated BV2 microglia in a dose-dependent manner (Figs 3a–c and 4, *p* < 0.05). On the contrary, SCH had no significant effect on the release of all these pro-inflammatory cytokines and NO (Figs 3 and 4, *p* > *0.05*). These results demonstrated that SCH had inhibitory effects against pro-inflammatory cytokine production in activated BV2 microglia.

### Effects of SCH on Nuclear NF-κB and IκB Activation and its Protein Levels in LPS-Treated BV2 Microglia

**Figure 5:**
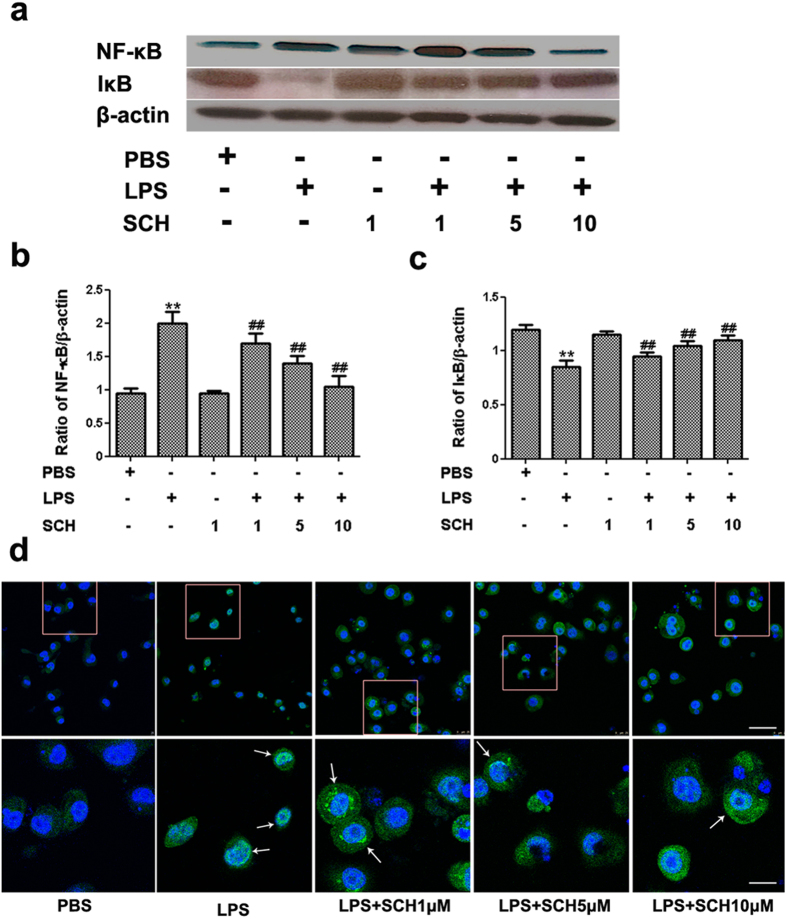
Effects of SCH on NF-κB and IκB expression in LPS-stimulated and SCH-treated BV2 cells. LPS-induced BV2 microglial cells were treated with different concentrations of SCH for 24 hr. (**a**) The levels of NF-κB and IκB were detected by Western Blotting. β-actin was used as an internal control. Each blot was representative of at least three independent experiments. **p < *0.001* versus control; ^##^*p* < *0.01* versus LPS (1 μ/ml) group. (**b**) The density of corresponding bands in (A) were quantitated and plotted as the ratio of NF-κB /β-actin. (**c**) The density of corresponding bands in (A) were quantitated and plotted as the ratio of IκB /β-actin. (**d**) Immunofluorescence result for p-NF-κB (green) in LPS-stimulated and SCH-treated BV2 cells. Scale bar = 200 μm. Magnification of 5x within the white rectangles in image below. p-NF-κB located in or around the nucleus by white arrows. Scale bar = 40 μm. Full-length blots are presented in Supplementary Fig. S1.

Accumulating evidence has highlighted the crucial role of NF-κB as a primary mediator of the gene network in the production of pro-inflammatory cytokines and enzymes involved in the process of inflammation^26^. As a transcription factor, NF-κB is activated by the phosphorylated IκB and translocated to the nucleus^27^. In this study, we also tested the level of NF-κB in LPS-stimulated and SCH-treated BV2 microglia. As shown in Fig. 5a–c(also Supplementary Fig. S1), (*p* < *0.05*), the level of NF-κB in BV2 microglia significantly increased and the level of IκB significantly decreased following exposure to LPS for 24 hr. In addition, SCH treatment significantly attenuated the nuclear translocation of NF-κB and degradation of IκB in a dose-dependent manner. To test for the presence of phosphorylated NF-κB (p-NF-κB), we stained the translocation of NF-κB with immunofluorescence. Our results showed that p-NF-κB (Green) located in or around the nucleus, and the SCH significantly inhibited NF-κB phosphorylation following LPS stimulation (Fig. 5d). These results suggest that SCH confers an anti-inflammatory effect by suppressing NF-κB activation in LPS-induced BV2 microglia.

### Effect of SCH on PC12 Cell Viability and Apoptotic Pattern Treated with Conditioned Medium from LPS-Stimulated BV2 Microglia

**Figure 6:**
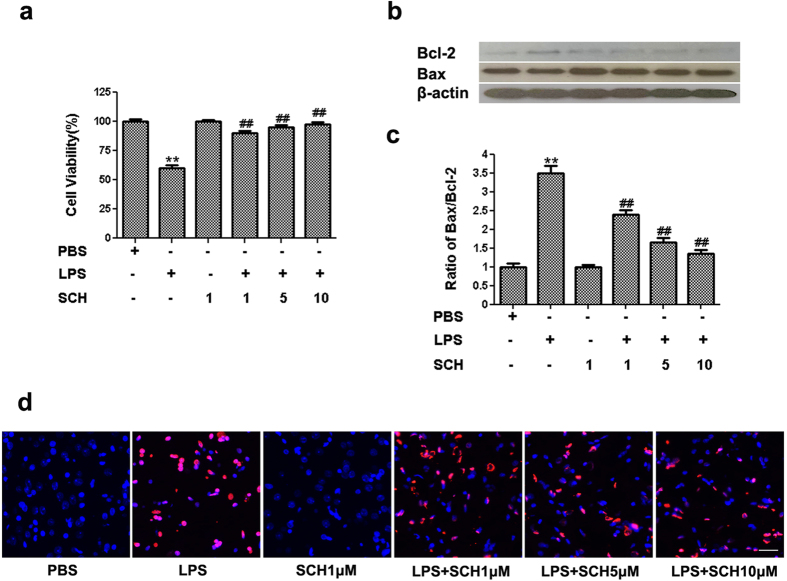
Effects of the conditioned medium from LPS-stimulated and SCH-treated BV2 microglia on PC12 cells . (**a**) After treatment of the conditioned medium for 24 hr, cell viability was measured by the MTT assay. (**b**) Bax, Bcl-2 and β -actin was detected by We stern blotting analysis. (**c**) The density of corresponding bands in (B) were quantitated and plotted as the ratio of Bax/Bcl-2. (**d**) Apoptotic cells induced by the conditioned medium from LPS-stimulated and SCH-treated BV2 cells. Detection of apoptotic cells by Tunel method. Scale bar = 200 μm. Results were expressed as mean ± SD (n = 3). **p < *0.001* versus control; ^##^*p* < *0.01* versus LPS (1 μg/ml) group. Full-length blots are presented in Supplementary Fig. S2.

To investigate the effects of LPS-stimulated BV2 microglia on PC12 cells, the PC12 cells were exposed to the microglia-conditioned media, according to a previously described protocol^28^. Cell viability was assessed using an MTT assay. Results showed that treatment of PC12 cells with conditioned medium harvested from LPS-stimulated BV2 microglia markedly decreased cell viability of PC12 cells to 61.0 ± 0.2%. When compared with PC12 cells treated with conditioned media from LPS-stimulated BV2 microglia, cell viabilities of those treated with various concentrations of SCH were increased significantly (*p* < *0.001*). These results suggest that the neurotoxic effects of condition media from LPS-stimulated BV2 microglia were reduced by SCH pretreatment in a dose-depend manner (Fig. 6a).

Furthermore, we detected the changes of pro-apoptotic protein Bax and anti-apoptotic protein Bcl-2 which are considered primary regulators of mitochondrial depolarization. Conditioned media from LPS-stimulated BV2 microglia was found to increase the ratio of Bax to Bcl-2. Pretreatment with SCH down-regulated Bax and up-regulated Bcl-2 protein level, leading to the decrease in the ratio of Bax to Bcl-2 in a dose-dependent manner (Fig. 6b,c, Supplementary Fig. S2, *p* < *0.05*).

Next, we observed the effects of conditioned media from LPS-stimulated and SCH-treated BV2 microglia on PC12 cell death using the TUNEL assay. We found that conditioned media from LPS-stimulated BV2 microglia increased PC12 cells death, while SCH attenuated LPS-induced cells death in a dose-dependent manner (Fig. 6d).

Taken together, these results indicate that SCH facilitates the protective effect on PC12 cells from death caused by neurotoxicity of LPS-stimulated BV2 microglia.

### PI3/Akt Pathways Mediate the Protective Effects of BV2-Microglia-Conditioned Media on PC12 Cells

**Figure 7:**
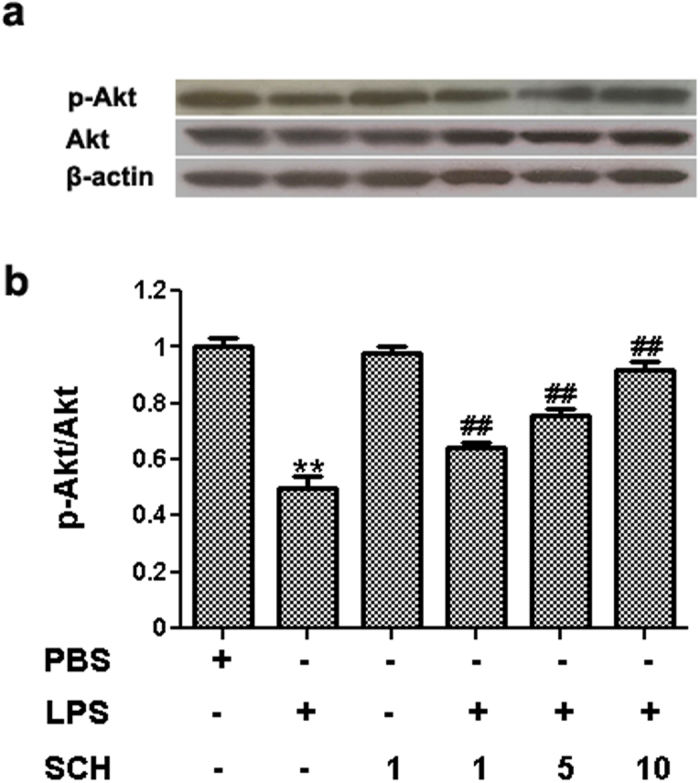
Expression pattern of p-Akt was detected by western blottling (**a**) and densitometric analysis in conditioned medium from LPS-stimulated and SCH-treated BV2 cells on PC12 neurons (**b**). Results were expressed as mean ± SD (n = 3). **p < *0.001* versus control; ^##^*p* < *0.01* versus LPS (1 μg/ml) group. Full-length blots are presented in Supplementary Fig. S3.

**Figure 8:**
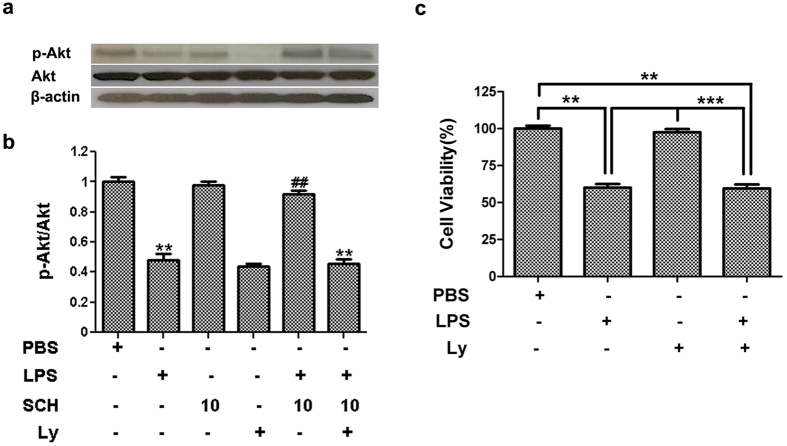
Expression pattern of p-Akt was detected by western blottling (**a**) and densitometric analysis in conditioned medium from LPS-stimulated and SCH-treated BV2 cells on PC12 neurons, in absence or in presence of Ly (10 μM) (**b**). Viability of PC12 cells after exposure to conditioned medium with different reagents (**c**). Results were expressed as mean ± SD (n = 3). **p < *0.001* versus control; ^##^*p* < *0.01* versus LPS (1 μg/ml) group; ***p < *0.001* versus Ly group. Full-length blots are presented in Supplementary Fig. S4.

PI3K/Akt pathway is known to be a pro-survival cell signaling pathway^29^, so we next investigated whether the protective effect of SCH on PC12 cells was mediated through this pathway. As shown in Fig. 7a,b (also Supplementary Fig. S3), Akt phosphorylation was significantly decreased after treatment with conditioned media from LPS-stimulated BV2 microglia, and SCH treatment significantly increased Akt phosphorylation in a dose-dependent manner in cultured PC12 cells, with no change in total Akt levels. The PI3K inhibitor Ly blocked those Akt responses, confirming the role of the pathway in this process (Fig. 8a,b, Supplementary Fig. S4).

To investigate the combination effects of LPS with Ly on the viability of PC12, the MTT assay was performed. As shown in the following graph (Fig. 8c), LPS with Ly together had no effect on PC12 cell viability compare to LPS treatment. When compared with PC12 cells treated with Ly, cell viability treated with LPS and Ly together was decreased significantly (*p* < *0.001*). The same trend of cell viability can be observed in LPS with Ly together and LPS alone compare to control group (*p* < *0.001*).

### PI3K/Akt Involvement in LPS-Stimulated and SCH-Treated BV2 Microglia on PC12 Neurons

**Figure 9:**
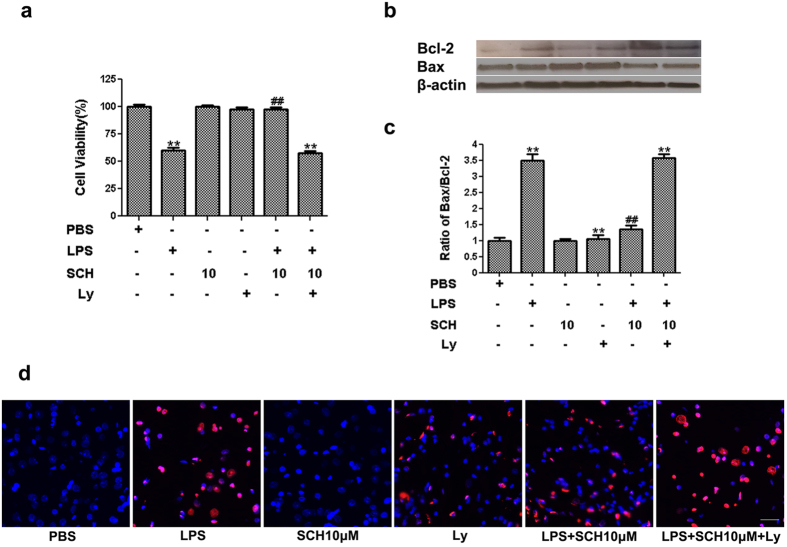
Involvement of PI3K/Akt in the effects of the conditioned medium from LPS-stimulated and SCH-treated BV2 cells on PC12 neurons. (**a**) After treatment of the conditioned medium for 24 hr, cell viability was measured by the MTT assay. (**b**) Bax, Bcl-2 and β -actin was detected by We stern blotting analysis. (**c**) The density of corresponding bands in (B) were quantitated and plotted as the ratio of Bax/Bcl-2. (**d**) Apoptotic cells induced by the conditioned medium from LPS-stimulated and SCH-treated BV2 cells, in absence or in presence of Ly (10 μM). Detection of apoptotic cells by Tunel method. Scale bar = 200 μm. Results were expressed as mean ± SD (n = 3). **p < *0.001* versus control; ^##^*p* < *0.01*versus LPS (1 μg/ml) group. Full-length blots are presented in Supplementary Fig. S5.

To establish a causal relationship, we further investigated whether blockade of Akt signaling in PC12 cells could negate the protective effects of SCH-treated BV-microglia-conditioned media. As shown in Fig. 9 (also Supplementary Fig. S5), the PC12-protective effects of 10 μM SCH were significantly reduced by pretreatment of Ly as measured by the cell viability and death assays (Fig. 9a–d). In addition, we found that the Ly pretreatment alone caused a slight increase in cell death in PC12 cells (Fig. 9b–d). The above results indicate that the PI3K/Akt pathway is involved in microglial inflammatory signaling and microglia-mediated neuronal cell death.

## Methods

### Materials

BV2 microglial cell line and PC12 cells were obtained from the cell bank of the Shanghai Institute of Cell Biology and Biochemistry, Chinese Academy of Sciences (Shanghai, China) and were grown in Dulbecco’s Modified Eagle Medium (DMEM), fetal bovine serum (FBS), penicillin/streptomycin from Invitrogen (Carlsbad, CA, USA). Antibodies against PAR-1, CD11b, Bax, Bcl-2, Akt, phospho-Akt, nuclear factor NF-κB p65, and IκBα were purchased from Santa Cruz Biotechnology (Santa Cruz, CA, USA). The fluorescent dye-conjugated secondary antibodies were purchased from Zymed (San Francisco, CA, USA). The PAR-1 antagonist SCH dihydrochloride was obtained from Tocris Bioscience (Bristol, UK). PI3K inhibitor Ly was purchased from Calbiochem (La Jolla, CA, USA). All other reagents were purchased from Sigma (St. Louis, MO, USA), or as otherwise indicated.

### Cell Culture and Treatment

BV2 microglia cells were maintained in DMEM supplemented with 10% fetal bovine serum, 100 U/ml penicillin and 100 μg/ml streptomycin in a humidified 5% CO_2_/95% air environment at 37 °C. All experiments were carried out 24 hr after the cells were seeded. The cells were treated with SCH diluted in DMSO with the final concentrations being 1, 5, 10 μM, and the cytotoxicity of SCH was evaluted by MTT in the following expriment, and LPS (1 μg/ml, Sigma-Aldrich, St. Louis, MO, USA) at the same time for 24 hr. The supernatants were removed and replaced with fresh DMEM for another 24 hr. Then BV2 cells were harvested for experiments. The PC12 cells were grown and maintained in DMEM containing 10% fetal bovine serum, 10% horse serum, 100 U/mL penicillin, and 100 μg/mL streptomycin at 37 °C under 5% CO_2_ atmosphere. The cells were passaged before reaching confluence using 0.025% (v/v) trypsin/EDTA in PBS. For the differentiation of PC12 cells, the cells were seeded on a collagen-coated dish at a density of 2 × 10^4^ cells/cm^2^. Then, the cells were exposed to 50 ng/mL NGF in medium containing 1% horse serum for 24 hr and the medium was routinely replaced every 2 days with fresh DMEM containing 50 ng/mL NGF and 1% horse serum until 6 days after the induction of differentiation. The conditions for PC12 cell differentiation were used in the following experiments. 10 μM Ly was applied to differentiated PC12 cells 30 min before condition media treatment. The BV2-conditioned medium of BV2 cells was removed and added to differentiated PC12 neurons, which were cultured in a humidified 5% CO_2_/95% air environment at 37 °C for 24 hr.

### Enzyme-Linked Immunosorbent Assay (ELISA)

The supernatant collected from BV2 microglia treated with LPS and SCH for 24 hr was measured for production of pro-inflammatory cytokines using ELISA. BV2 microglia were cultured at the concentration of 10^6^/well in a 6-well plate. The concentrations of pro-inflammatory cytokines released into the culture medium were measured using mouse IL-1β, IL-6, TNF- α, and IL-10 ELISA kits (BD Biosciences, San Jose, CA, USA). The assays were carried out according to the manufacturer’s protocol and the absorbance was read at a wavelength of 450 nm using a microplate reader (Model 550, Bio-Rad, Hercules, CA, USA).

### NO Quantification

Concentrations of LPS-stimulated nitric oxide (NO) released from BV2 microglia were determined using the Griess reagent as previously reported^30^. Supernatants were mixed with the same amounts of Griess reagent. Samples were incubated at room temperature for 10 min and optical density was measured at 540 nm using a microplate reader (Model 550, Bio-Rad, Hercules, CA, USA).

### MTT Cell Viability Assay

Cell viability was measured using MTT [3-(4,5-dimethylthiazol-2-yl)-2,5-diphenyl tetrazolium bromide] assay. BV2 cells and PC12 neurons were plated at a density of 4.5 × 10^3^ and 2 × 10^3^ cells per well respectively in 96-well plates. Specifically, PC12 was treated by the conditioned medium separated from BV2 cells for 24 hr. MTT solution at the final concentration of 5 mg/mL was added to each well and plates were incubated for another 4 hr. Each well was then aspirated and 150 ul DMSO (dimethyl sulfoxide) was added. Finally, absorbance was read at 540 nm using a microplate reader (Bio-Rad, Hercules, CA, USA).

### Western Blotting Assay

Western blot analysis was implemented to explore the expressions of Bax, Bcl-2, NF-κB p65, IκBα, Akt and phospho-Akt, and β-actin. After washing in TBST, immunoblots were incubated with horseradish peroxidase-conjugated secondary antibodies (Cell Signaling Technology, Danvers, MA, USA) for 1 hr. The immunoblots were developed with normal or enhanced chemiluminescence (ECL) reagents (Millipore, USA), and measured with Quantity Software (Bio-Rad, Hercules, CA, USA).

### Immunofluorescence

After the BV2 microglia were seeded on slices for 24 hr, the cells were treated with SCH diluted in DMSO with the final concentrations being 1, 5, 10 μM, respectively, and also with LPS at 1 μg/mL simultaneously for 24 hr. After the supernatant was removed, slices were subject to immunostaining analysis. Cells were fixed in 4% paraformaldehyde for 30 min at room temperature and washed three times with PBS. Non-specific binding was blocked with 3% bovine serum, and treated with 0.1% Triton X-100 in 1% BSA-PBS for 15 min. Being washed, After a wash, specific primary antibodies were used against CD11b and PAR-1. For double immunofluorescence experiments, cells were incubated with FITC-conjugated anti-rabbit IgG and/or TRITC-conjugated anti-goat IgG secondary antibody. Subsequently, slices were incubated at 4 °C overnight with monoclonal mouse primary NF-κB p65 antibody, and then washed and incubated with a goat anti-rabbit IgG for 2 hr in a dark room. The nucleus was stained using DAPI for 20 min. Control climbing slices were run following identical protocols, but omitting the primary antibodies. For each group, three cell climbing slices were examined in a blinded fashion.

### *In Situ* Cell Death Detection

PC12 neurons were observed to measure how conditional medium from BV2 microglia cultured cells affected death using a TUNEL method. In brief, PC12 neurons from different treatment groups were stained using an *in situ* apoptotic cell death detection kit, TMR red (Roche Molecular Biochemicals, Indianapolis, USA). All preparations of solutions and buffers were performed following the manufacturer’s instructions and the TUNEL reaction mixture was prepared immediately before use. After counterstaining with DAPI, fluorescein labels incorporated in the nucleotide polymers were detected and photographed by fluorescence microscopy.

### Statistical Analysis

All procedures stated above were repeated at least three times in independent experiments, and all data was described as mean ± SD and were analyzed using SPSS 11.0 statistical software (SPSS Inc., Chicago, IL, USA). To contrast the effect of SCH on pro-inflammatory responses, the concentration of pro-inflammatory factors, the level of immunofluorescent densities, and the densitrometric measurements of above mentioned parameters were compared between groups treated with respective doses of SCH (1, 5, and 10 μM) with the control group defined as treated by LPS only. Another baseline control group defined as PBS treated only was also added. Furthermore, to associate the effect of SCH with the p-Akt/Akt pathway, an additional reagent Ly was added to observe the negative effect of Ly on SCH in respect to p-Akt/Akt levels. The dose of 10 μM for SCH was chosen according to preliminary results to maximize the effect of SCH on inflammation inhibition. All statistical comparisons across groups for continuous variables were performed using a one-way ANOVA, with significance level defined as *p* < 0.05. All post-hoc tests were conducted using Tukey’s multiple comparison test.

## Discussion

Previous reports indicate high expression of PAR-1 at both neuronal and glial cells, including neurons, astrocytes, oligodendrocytes, and microglia. PAR-1 has also been shown to be involved in several brain pathologies, such as Parkinson’s disease, Alzheimer’s disease, multiple sclerosis, stroke, and human immunodeficiency virus-associated dementia^31^,^32^,^33^, suggesting its potential role in the regulation of brain function and the pathogenesis of these pathological changes.

Our study demonstrated that the selective PAR-1 antagonist SCH suppresses excessive microglial activation and subsequent neuronal death in a cell culture-based neuroinflammation model. In addition, we have shown that suppression of PI3K/Akt eliminates the neuroprotective effect through inhibition of PAR-1. Collectively, this data suggests that inhibition of PAR-1 by SCH provides protection against neurotoxic effects induced by excessive inflammatory activation in neural cells via certain mechanisms involving the activation of PI3K/Akt pathway.

Microglia are considered the first line of defense in the innate CNS immune response, crucial for neuroprotective and repair processes. However, abnormal activation of microglia induces a number of major cellular responses in the pathogenesis of inflammatory responses, leading to cell death or apoptosis of neural cells^8^. These effects are mediated by the expression of numerous proinflammatory factors, including NO, IL-6, IL-1α, IL-1β, and TNF-α *in vitro* and *in vivo*^31^. The expression of the immuno-suppressive cytokine IL-10 has also been detected in rat microglia, and has been shown to inhibit TNF-α release^34^. These actions are partially regulated by the NF-κB signaling pathway, which has been proposed to play an essential role in host defense and inflammatory responses to extracellular stimuli^35^.

PAR-1 is a protease receptor located on the surface of microglia and that activates an immune response upon binding to its ligand^36^. Provided it has been known to suppress inflammation in many disease processes, including endotoxin shock, Parkinson’s disease and SAH^37^,^38^,^39^, some of which are mediated through PAR activation^40^, it is not surprising that the suppression of microglial cell PAR-1 has neuroprotective effects. However, the role of this major thrombin receptor^36^, PAR-1, in inflammation after brain injury is not clear. In conjunction with previous studies, our results suggest that all cultured BV2 microglia cells show strong immunoreactivity of PAR-1. Subsequently, to address the possible involvement of PAR-1 in LPS-induced microglial activation and the anti-inflammatory role of SCH, BV2 cells were pretreated with PAR-1 inhibitors SCH and then treated with LPS, which was considered as a potent experimental tool for microglial activation and has been implicated in brain injury and neurodegeneration^41^. The inhibition of PAR-1 was found to significantly suppress the expression of IL-6, IL-1β, TNF-α and NO production, and prevent the activation of NF-κB in BV2 cells. In addition, the inhibition of PAR-1 by SCH attenuated the decrease in cell viability and protected PC12 neurons from death induced by the conditioned medium from LPS-stimulated BV2 cells.

Interestingly, in concentrations of 1, 5, and 10 μM, we found SCH exerted its protective role in a dose-dependent manner. This phenomenon led us to hypothesize that SCH may interact with proteins in the NF-κB pathway. Studies confirm that NF-κB can be activated by a series of stimuli^42^, and that activated NF-κB can be translocated from the cytoplasm to the nucleus where it interacts with κB elements in the promoter region of a variety of inflammatory response genes, and activates their transcription^43^. In spite of previous reports consistent with our results, the molecular mechanism underlying neuroprotective activity of SCH still remains to be elucidated.

Evidence shows that IκB degradation is mediated via phosphorylation of IκB kinase by Akt in activated microglia in several types of cells^44^. Therefore, we further investigated the PI3K/Akt pathway, which is known to be pro-survival cell signaling pathway. Indeed, we demonstrated here that PI3K/Akt mediated pathways were provoked by SCH treatment in PC12 cells. This protective effect may be mediated in part via Akt signaling pathway. In brief, microglia-conditioned media up-regulated the phosphorylation levels of Akt in PC12 cells, with no change in total Akt levels. Blockade of Akt signaling with Ly diminished the protective effects of microglia-conditioned media.

Taken together, our findings demonstrate that PAR-1 is involved in LPS-induced microglial activation, and appears to switch off the inflammatory cascade. However, there are several limitations to our study that need to be addressed. First, BV2 cell line is the most frequently used alternative *in vitro* models for microglia activation. There is an explosion of work on microglial research has been performed using cell lines such as N9^45^ and BV2 cell lines^46^. Previous reported that BV2 cell lines exhibit many similar characteristics with primary microglia both *in vitro* and vivo model^47^,^48^. However, doubt has been recently raised in regard to the value of BV2 cell lines as a model system. Butovsky *et al*. demonstrated that adult primary microglia expressed a unique molecular signature that was totally different from the BV2 cell lines^49^. Furthermore, immortalization renders BV2 cell lines some ways different from primary microglia in culture or in the CNS^50^. thus, the characterization of phenotypic activation on how closely using a specific cell line resemble primary microglia still needs to study further. In addition, it is unclear what the underlying mechanism for PAR-1 activation of PI3/Akt is. As a strong PI3K inhibitor^51^, the effect of Ly provide insights into a possible link between the PI3K activation pathway and cell death regulation. However, there are potential possibilities that mutiple proteins are also involved in this process. Hence, another well-characterized ‘orthogonal’ chemical probe having a completely different chemical structure is needed to reduce the probability of off-targets for further study, and the additional contributing components in this signaling pathway that contribute to PC12 cell protection remain to be investigated. Secondly, we cannot exclude the possible involvement of other PAR series receptors, including PAR-2, PAR-3 or PAR-4, which are widely expressed in the brain, including neurons, microglia, astrocytes, and oligodendrocytes^33^. Thirdly, due to limited accessibility of PAR-1 antagonists, the selection of SCH was solely based on the fact that previous literature has described it as highly potent and specific^52^. Previous studies show that the inhibition of inflammatory reactions by the PAR-1 antagonist SCH was incomplete as compared with a broad spectrum PAR inhibitor cathepsin G^53^. The role of the series of PAR receptors in this inflammatory reaction and the mechanisms involved remain to be further elucidated.

In summary, this study uncovered the suppressive effect of SCH on LPS-induced excessive microglial activation and the subsequent neuronal cell death via up-regulation of Akt-mediated pathways. The beneficial effects of SCH could be linked to its ability to block the inflammatory response and subsequently inhibit the death of PC12 neurons from the cytotoxicity of activated BV2 cells. These neuro-protective effects appear to be related to the inhibition of PAR-1, suggesting that a PAR-1 antagonist has important potential for development into a novel neuroprotective strategy for pharmaceutical intervention of neuroinflammation-related diseases.

## Additional Information

**How to cite this article**: Li, Y. *et al*. Interference with Protease-activated Receptor 1 Alleviates Neuronal Cell Death Induced by Lipopolysaccharide-Stimulated Microglial Cells through the PI3K/Akt Pathway. *Sci. Rep.* **6**, 38247; doi: 10.1038/srep38247 (2016).

**Publisher's note:** Springer Nature remains neutral with regard to jurisdictional claims in published maps and institutional affiliations.

## Supplementary Material

Supplementary Information
