# Supplementary material for: Interference with Protease-activated Receptor 1 Alleviates Neuronal Cell Death Induced by Lipopolysaccharide-Stimulated Microglial Cells through the PI3K/Akt Pathway
Source: Sci Rep. 2016 Dec 2;6:38247. doi: 10.1038/srep38247 (PMC5133627; doi:10.1038/srep38247)
Supplement: Supplementary Information [file srep38247-s1.pdf]

Supplemental Information

Interference with Protease-activated Receptor 1 Alleviates Neuronal Cell Death Induced by Lipopolysaccharide-Stimulated Microglial Cells through the PI3K/Akt Pathway

Authors: Yuxin Li, M.D.,[1,2*] Wuyang Yang, M.D., M.S.,[2] Alfredo Quinones-Hinojosa, M.D., [2] Baocheng Wang, M.D., [1] Shujun Xu, M.D., [3] Weijie Zhu, M.D., [1] Feng Yu, M.D., [1] Shaoji Yuan, M.D., [1] Peigang Lu, M.D. [1*]

Affiliations:

[1] Department of Neurosurgery, Jinan General Hospital of PLA, Jinan, Shandong, 250031, P.R. China

[2] Department of Neurosurgery, Johns Hopkins University School of Medicine, Baltimore, MD, 21205, USA

[3] Department of Neurosurgery, Qilu Hospital, Shandong University, Jinan, Shandong, 250000, P.R. China

***Corresponding Author:** Department of Neurosurgery, Jinan General Hospital of PLA, 25 Shifan Road, Jinan, 250031, P.R. China. Tel: +86531 51665350; fax: +86531 51666603. Yuxin Li (E-mail: Yuxinli1999@outlook.com) and Peigang Lu (E-mail: Lupeigang912@126.com).

For figure 5-a

NF-κB

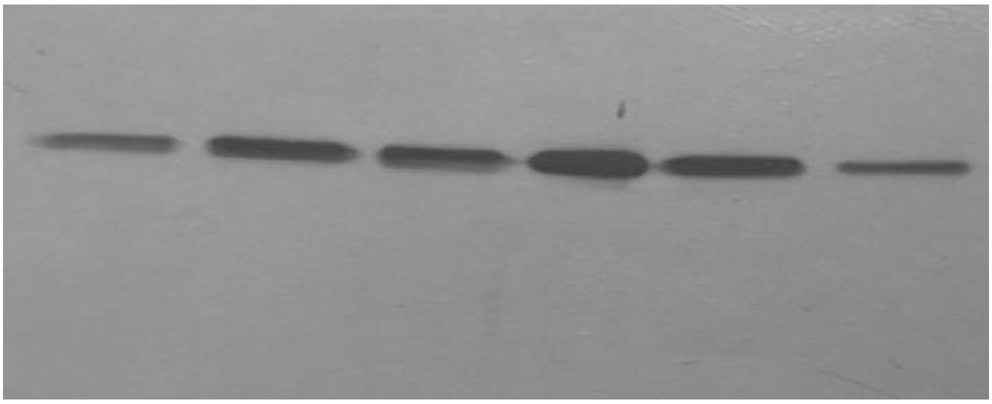

IκB

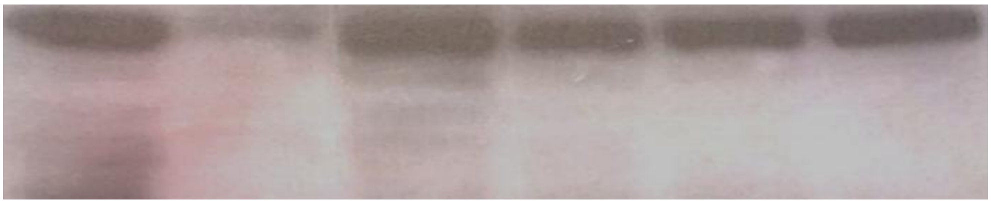

β-actin

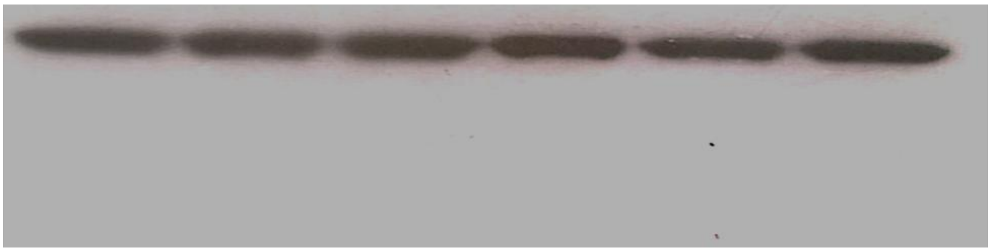

Supplemental Fig. S1. The original blots of Fig. 5 in the text.

For figure 6-b

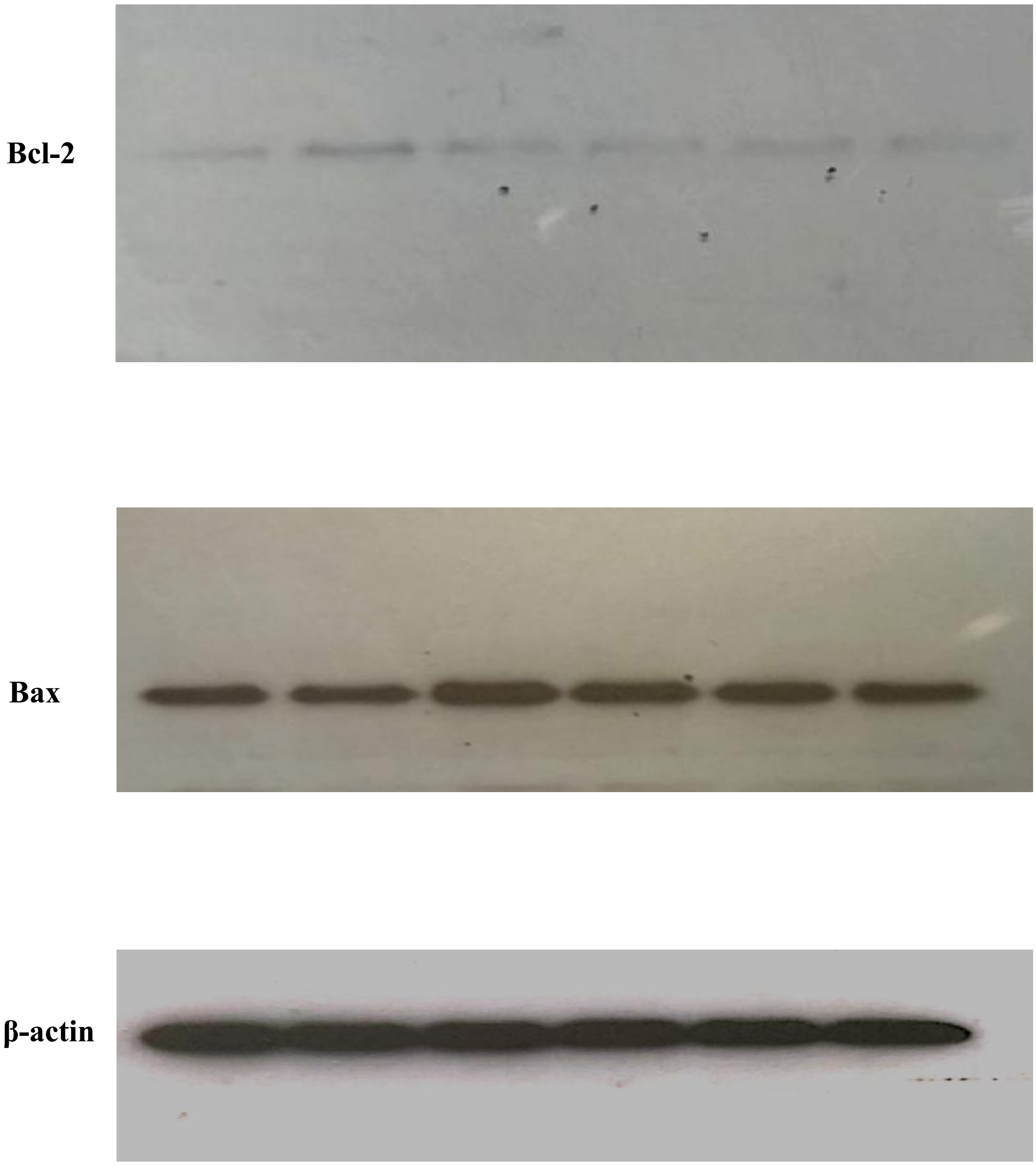

Supplemental Fig. S2. The original blots of Fig. 6 in the text.

For figure 7-a

p-Akt

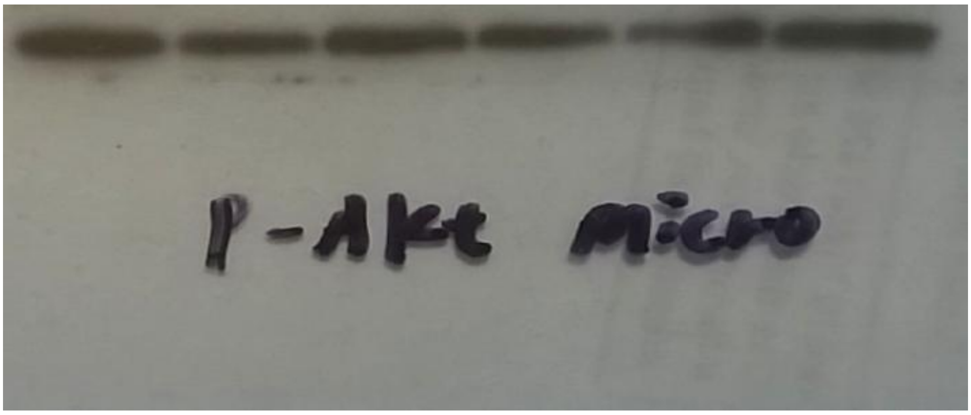

Akt

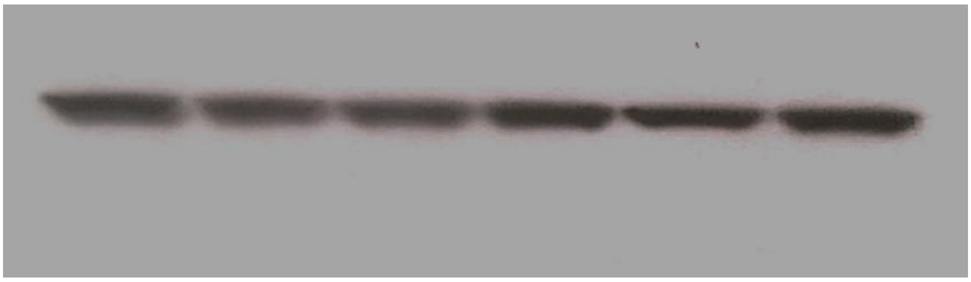

β-actin

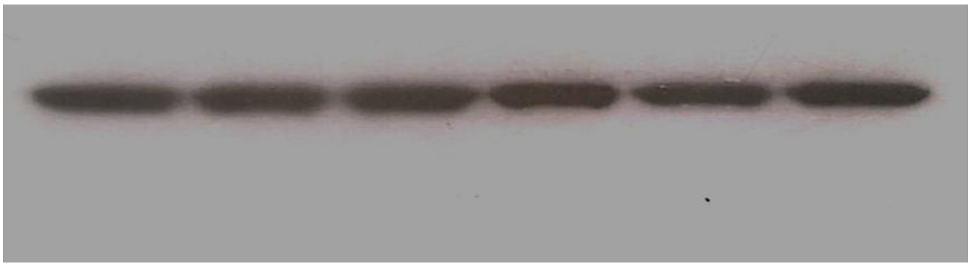

Supplemental Fig. S3. The original blots of Fig. 7 in the text.

For figure 8-a

p-Akt

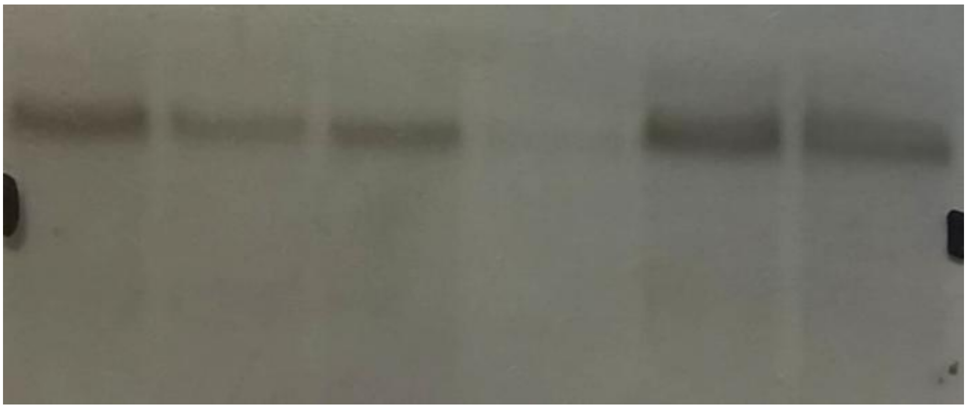

Akt

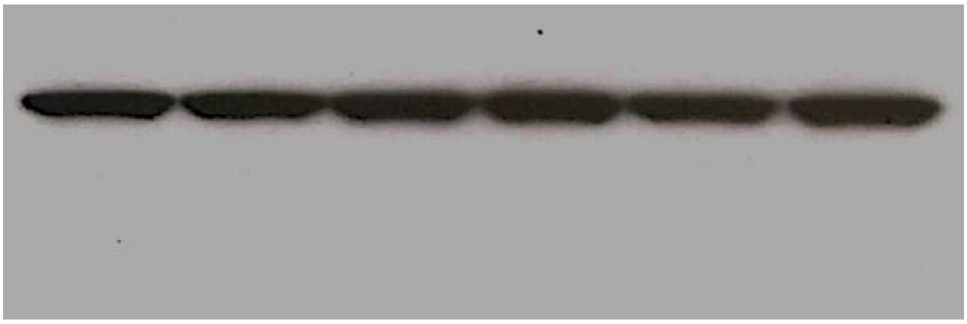

β-actin

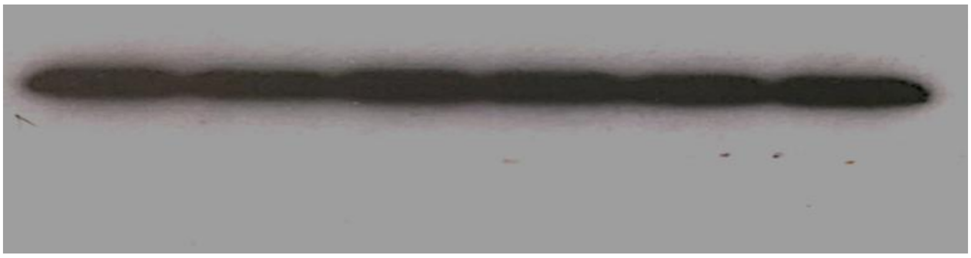

Supplemental Fig. S4. The original blots of Fig. 8 in the text.

For figure 9-b

Bcl-2

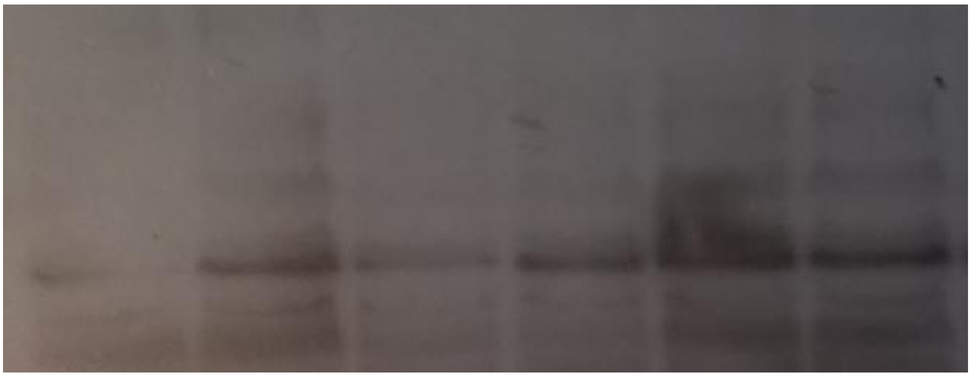

Bax

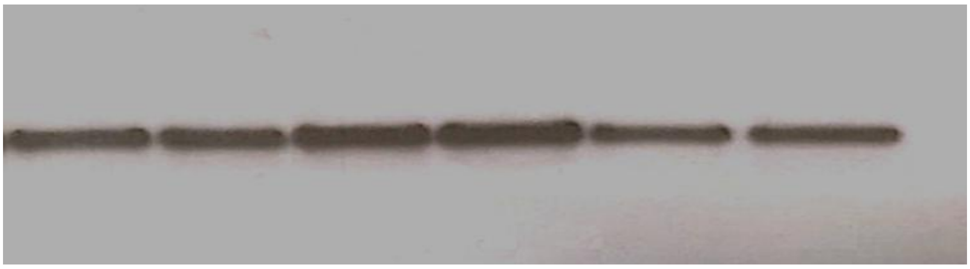

β-actin

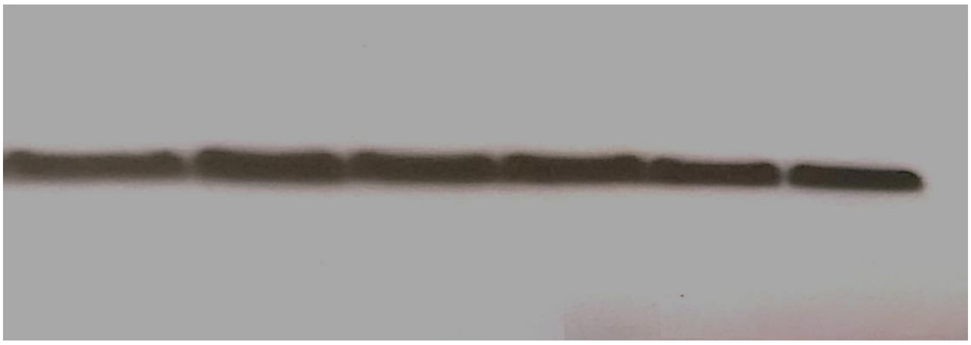

Supplemental Fig. S5. The original blots of Fig. 9 in the text.
